# Association between Airborne Fine Particulate Matter and Residents’ Cardiovascular Diseases, Ischemic Heart Disease and Cerebral Vascular Disease Mortality in Areas with Lighter Air Pollution in China

**DOI:** 10.3390/ijerph15091918

**Published:** 2018-09-03

**Authors:** Junfang Cai, Shuyuan Yu, Yingxin Pei, Chaoqiong Peng, Yuxue Liao, Ning Liu, Jiajia Ji, Jinquan Cheng

**Affiliations:** 1 National Institute of Environmental Health and Related Product Safety, Chinese Center for Disease Control and Prevention, Beijing 100021, China; caijunfang@nieh.chinacdc.cn; 2 Shenzhen Center for Disease Control and Prevention, Shenzhen 518055, China; shuyuanyu2008@163.com (S.Y.); pcq@szcdc.net (C.P.); lyxchinaren@163.com (Y.L.); liun@szcdc.net (N.L.); jiajia0929@sohu.com (J.J.); 3 CFETP, Chinese Center for Disease Control and Prevention, Beijing 100050, China; peiyingxin@hotmail.com

**Keywords:** air pollution, cardiovascular disease, ischemic heart disease, cerebral vascular disease, mortality, generalized additive model, time-series

## Abstract

Background: China began to carry out fine particulate matter (PM_2.5_) monitoring in 2013 and the amount of related research is low, especially in areas with lighter air pollution. This study aims to explore the association between PM_2.5_ and cardiovascular disease (CVD), ischemic heart disease (IHD) and cerebral vascular disease (EVD) mortality in areas with lighter air pollution. Methods: Data on resident mortality, air pollution and meteorology in Shenzhen during 2013–2015 were collected and analyzed using semi-parametric generalized additive models (GAM) with Poisson distribution of time series analysis. Results: Six pollutants were measured at seven air quality monitoring sites, including PM_2.5_, PM_10_, SO_2_, NO_2_, CO and O_3_. The PM_2.5_ daily average concentration was 35.0 ± 21.9 μg/m^3^; the daily average concentration range was from 7.1 μg/m^3^ to 137.1 μg/m^3^. PM_2.5_ concentration had significant effects on CVD, IHD and EVD mortality. While PM_2.5_ concentration of lag5 and lag02 rose by 10 μg/m^3^, the excess risk (ER) of CVD mortality were 1.50% (95% CI: 0.51–2.50%) and 2.09% (95% CI: 0.79–3.41%), respectively. While PM_2.5_ concentration of lag2 and lag02 rose by 10 μg/m^3^, the ER of IHD mortality were 2.87% (95% CI: 0.71–5.07%) and 3.86% (95% CI: 1.17–6.63%), respectively. While PM_2.5_ concentration of lag4 and lag04 rose by 10 μg/m^3^, the ER of EVD mortality were 2.09% (95% CI: 2.28–3.92%) and 3.08% (95% CI: 0.68–5.53%), respectively. Conclusions: PM_2.5_ increased CVD mortality. The government needs to strengthen the governance of air pollution in areas with a slight pollution.

## 1. Introduction

The 2006 World Health Organization Air Quality Guidelines recommend using particulate matter with an aerodynamic diameter of under 2.5 microns (fine particulate matter, PM_2.5_), rather than particulate matter with particle size below 10 microns (PM_10_), as an indicator of air particle concentration [1]. Among atmospheric pollutants, fine particulate matter (PM_2.5_) is consistently associated with adverse human health [2,3,4] and is of great concern to the general public. The majority of studies of PM_2.5_ and daily mortality have been conducted in North America and Europe, with a small number of studies in other regions of the world [5].

Environmental pollution is very serious in China. The Chinese government has paid more and more attention to the prevention and control of environmental pollution. The report of the 19th Session of National Congress of the Communist Party of China regards pollution prevention as one of the three major battles [6]. Governments at all levels attach great importance to environmental pollution control, especially in areas with serious pollution. Lighter polluted areas in China (although the pollution level is much higher than in Europe and America) still do not attract enough attention.

China began to carry out PM_2.5_ monitoring in 2013, and few studies have focused on this issue, especially in areas with lighter air pollution. “Lighter air pollution” is relative to most parts of China. Both the Chinese Environmental Status Bulletin and the National Air Quality real-time ranking show that Shenzhen has lighter air pollution than other cities in China [7,8]. The average annual concentration of PM_2.5_ in 272 cities in China from 2013 to 2015 nationwide was 56 μg/m^3^; North-east, North, East, Middle-South, South-west and North-west were 52 μg/m^3^, 69 μg/m^3^, 59 μg/m^3^, 56 μg/m^3^, 42 μg/m^3^ and 55 μg/m^3^, respectively [9]. At the same time, the PM_2.5_ annual average concentration in Shenzhen was 35 μg/m^3^. It shows that Shenzhen air pollution is relatively light in China.

Shenzhen is China’s first special economic zone. It is the window of China’s reform and opening up. It is one of the three largest national financial centers in China. In the “Economist” list of “The Most Economically Competitive Cities in the World” in 2012, Shenzhen ranked second. Shenzhen is situated in South-eastern China, 113°46–114°37 E and 22°27–22°52 N, with an area of 1991.64 km^2^, neighboring Hong Kong. Shenzhen has a sub-tropical maritime climate (warm temperatures, abundant rainfall). The annual average temperature is 23.5 °C. January and July monthly average temperatures were 16 °C and 28.9 °C, respectively. This study aims to explore the association between PM_2.5_ and cardiovascular disease (CVD), ischemic heart disease (IHD) and cerebral vascular disease (EVD) mortality in areas with lighter air pollution in China.

## 2. Materials and Methods

### 2.1. Materials

#### 2.1.1. Data on Resident Mortality

Data on resident mortality in Shenzhen during 1 January 2013–31 December 2015 (a total of 1095 days) were collected from Shenzhen Center for Disease Control and Prevention (CDC), Shenzhen Public Security Bureau and Shenzhen Funeral Home. Each record includes a variable identity card number, name, gender, age, date of birth, date of death, the main cause of death and the main cause of death International Classification of Diseases (ICD) coding. Data for the three agencies were compared in accordance with identity card number and name, and repetitive data excluded. There is not one death due to influenza in the database. Grouping the daily mortality of residents and sorting and screening the data according to the International Classification of Diseases Revision 10 (IDC-10), the data encoded as I00-I99 represent total CVD mortality, I20-I25 represent IHD mortality and I60-I69 represent EVD mortality.

#### 2.1.2. Air Quality Monitoring Data

**Figure 1:**
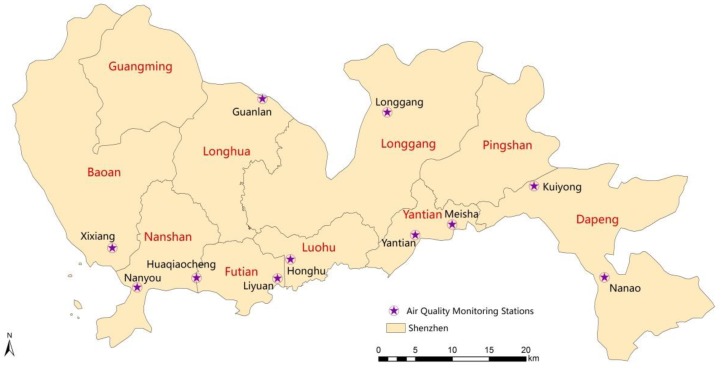
Distribution of state-controlled air quality monitoring sites in Shenzhen.

There were eleven air quality monitoring sites at the Shenzhen Environmental Monitoring Station. Seven of them were urban air quality monitoring sites. They were Xixiang, Nanyou, Huaqiaocheng, Honghu, Liyuan, Yantian and Longgang. Daily air quality monitoring data were provided by the seven urban air quality monitoring sites, covering the period of 1 January 2013–31 December 2015. The air quality monitoring data were based on the mean of daily average concentration values at the seven sites, including six indicators, namely: PM_2.5_, PM_10_, sulfur dioxide (SO_2_), nitrogen dioxide (NO_2_), carbon monoxide (CO) and ozone (O_3_). The other four were background monitoring sites and regional air quality monitoring sites. They were Guanlan, Meisha, Kuiyong and Nanao. Data from these four monitoring sites were not used. The distribution of monitoring sites was shown in Figure 1.

#### 2.1.3. Meteorological Data

There were forty regional meteorological observation stations in Shenzhen. Daily monitoring data from these stations was collected by the Shenzhen Meteorological Service Center. Meteorological data, which was collected from daily routine monitoring data, were released by the Shenzhen Meteorological Service Center, covering the period of 1 January 2013–31 December 2015. This data included daily average temperature, daily average relative humidity (RH), daily average atmospheric pressure and daily average wind speed.

### 2.2. Methods

#### 2.2.1. Basic Description

Resident mortality data, daily air pollutant data, and meteorological data were not normally distributed. The data are described by mean, standard deviation, minimum, Q1, Q2, Q3 and maximum in order to show the data more clearly.

#### 2.2.2. Analysis of Time Series

Resident mortality data were consolidated into the daily mortality of residents through grouping, screening and summary. The air pollutant variables, PM_2.5_ and CO, were based on the mean of 24 h average concentration values at the seven monitoring sites of the Shenzhen Environmental Monitoring Station. The air pollutant O_3_ variable was based on the means of 8 h sliding average concentrations. Multiple time series plots were produced with time on the horizontal axis and daily average concentrations of pollutants (PM_2.5_, CO and O_3_) on the vertical coordinate axes.

#### 2.2.3. Correlation Analysis

Correlation analysis was conducted using SPSS 19.0 statistical software (SPSS Inc., Chicago, IL, USA). Daily air pollutant data and meteorological data were not normally distributed; therefore, Spearman correlation analysis was conducted with environmental pollutants and meteorological factors. The greater the correlation coefficient between PM_2.5_ and other factors, the higher the probability of collinearity. In order to control confounding factors and avoid the effects of collinearity, meanwhile considering the effects of CO and O_3_ on cardiovascular disease, CO and O_3_ were controlled as confounding factors [10,11].

#### 2.2.4. Generalized Additive Models (GAM)

Resident mortality data typically followed a Poisson distribution. Analysis of time series was conducted by using semi-parametric GAM based on Poisson distribution [12,13,14,15,16,17]. The dependent variable is linked with the independent variable through log transformation [18,19,20]. Firstly, the basic model was constructed. Following the control of influence of long-term trend, seasonal trend, day of the week and public holiday, daily average temperature and daily average relative humidity were entered into the model [20,21]. For the basic model, air pollutants were not entered as independent variables; however, they were used in later models as described in the next paragraph. The long-term trend, daily average temperature and daily average relative humidity were adjusted by cubic regression spline function [22]. Seasonal trend, day of the week and public holiday were adjusted by dummy variables. The degrees of freedom (df) in previous literature were different, ranging from two to ten [9,23,24,25]. Some used the results of previous literature directly [26,27]. The optimal model was fitted to make the result more stable by changing df to a larger range (the long-term trend df from one to fourteen per year, the daily average temperature df from one to seven, and the daily average relative humidity df from one to seven). Based on the unbiased risk estimate (UBRE, UBRE is a readjustment standard of Akaike information criterion (AIC)) value and previous literature [28,29], the best parameters were established. For the basic regression model, we ultimately used seven df per year for the time variable, three df for the temperature variable, and three df for the humidity variable. Then, the single pollutant model was constructed. Single-day lag models underestimate the cumulative effect of pollutants on mortality [30]. Therefore, we built both single-day lag and multi-day lag models. PM_2.5_ concentration with different lags from 0 to 5 days prior to mortality (lag0–lag5) and moving averages from day 0 to day 1–5 prior to the mortality (lag01–lag04) were included in the model, respectively. Finally, two-pollutant models were constructed. We did not control SO_2_ and NO_2_ in the regression models because our Spearman correlation analysis showed that PM_2.5_ was highly correlated with SO_2_ and NO_2_. Previous literature showed that simultaneously entering SO_2_ (or NO_2_) into the models can bring unstable parameter estimates when the pollutants involved suffer high inter-correlation [31]. To examine the independent effects of PM_2.5_ on resident mortality, CO and O_3_ were controlled in the regression models respectively because previous literature and our Spearman correlation analysis showed that the correlation between CO and PM_2.5_, and O_3_ and PM_2.5_, were low [32]. CO and O_3_ were included in the regression models respectively with the same lag (0–5 days) prior to mortality (lag0–lag5) and moving averages from day 0 to day 1–5 prior to the mortality (lag01–lag04). Two-pollutant models were constructed respectively. The daily average concentration of pollutants PM_2.5_, CO and O_3_ were used as continuous variables to enter the model. The excess risk (ER, ER = (e^β*10^ − 1)) of resident mortality with different health effects, as well as its 95% CI value, were calculated using regression coefficients and standard errors along with the incremental increase of 10 μg/m^3^ in PM_2.5_ concentration. Quantitative analysis of the short-term effect of PM_2.5_ upon resident mortality was conducted [21]. The determined model expression is as follows:

*Log*[*E*(*y_t_*)] = *β*X*_t_* + *s*(*time*,*df*_1_) + *as*.*factor(season)* + *as*.*factor*(*dow*) + *as*.*factor*(*holiday*) + *s*(*temperature*,*df*_2_) + *s*(*humidity*,*df*_3_) + *α*

Here *y_t_* represents the number of mortalities at day *t*; *E*(*y_t_*) represents the expected number of mortalities at day *t*; *β* is the linear regression coefficients estimated by the GAM; X*_t_* indicates the concentration of pollutants at day *t*; *s* represents the nonparametric smoothing function; *df*_1_ is the degree of freedom for adjusting long-term trend in the nonparametric function; *season* is the dummy variable for season; *dow* and *holiday* are the dummy variables for day of the week and holiday, respectively, used to control the short-term fluctuations in the number of daily resident mortality; *df*_2_ and *df*_3_ are the degrees of freedom for adjusting the nonparametric smoothing function daily average temperature and daily average relative humidity; *α* is the residual error [22]. Resident mortality data, air quality monitoring data and meteorological data have no missing values. The pollutant concentration had missing values when analyzing the lag effect. The missing values were processed by deleting the corresponding record. Effect estimates were considered statistically significant if *p* < 0.05, and borderline significant if *p* < 0.10 [33]. Specific *p* values are listed. Statistical analysis was done by R3.2.0 software (open source software, Auckland, New Zealand). The GAM was constructed by the penalized splines function of GAM in the mgcv package.

## 3. Results

### 3.1. Basic Information of the Death of Residents from CVD

A total of 14,537 people died due to CVD; 3–32 people died due to CVD every day; the average age of CVD death was 67.4 years old, and the age range was 0–109 years old. In residents that died from CVD, 3,188 people died because of IHD, accounting for 21.9% of the total CVD deaths; 0–14 people died due to IHD every day; the average age of IHD death was 66.6 years old, and the age range was 0–109 years old. EVD accounted for 4,028 deaths, which was 27.7% of the total CVD deaths; 0–13 people died due to EVD every day; the average age of EVD death was 65.9 years old, and the age range was 0–104 years old. See Table 1 for the basic information of death residents from CVD in Shenzhen (2013–2015).

### 3.2. Information of Air Pollutants and Meteorological Factors

In 2013–2015, the PM_2.5_ annual average concentrations in Shenzhen were 40.2 μg/m^3^, 34.7 μg/m^3^ and 30.1 μg/m^3^, respectively; the mean of the PM_2.5_ daily average concentration was 35.0 μg/m^3^, the daily average concentration range was from 7.1 μg/m^3^ to 137.1 μg/m^3^, exceeding the national Grade 1 criterion of China (concentration limit < 35 μg/m^3^) on 458 days [34], and exceeding the national Grade 2 criterion of China (Concentration limit < 75 μg/m^3^) on 58 days. NO_2_ annual average concentration exceeded the national criterion of China (concentration limit < 40 μg/m^3^). PM_10_ and SO_2_ annual average concentration reached the national Grade 2 (concentration limit: PM_10_ < 70 μg/m^3^, SO_2_ < 60 μg/m^3^) criterion and Grade 1 (concentration limit: PM_10_ < 40 μg/m^3^, SO_2_ < 20 μg/m^3^) criterion of China, respectively. See Table 2 for a summary of air pollutants and meteorological indicators. See Table S1 for People’s Republic of China Ministry of Environmental Protection, Atmospheric environmental quality standards (GB3095-2012).

### 3.3. Time Series Chart on Resident Mortality from CVD versus Air Pollutant Concentration

**Figure 2:**
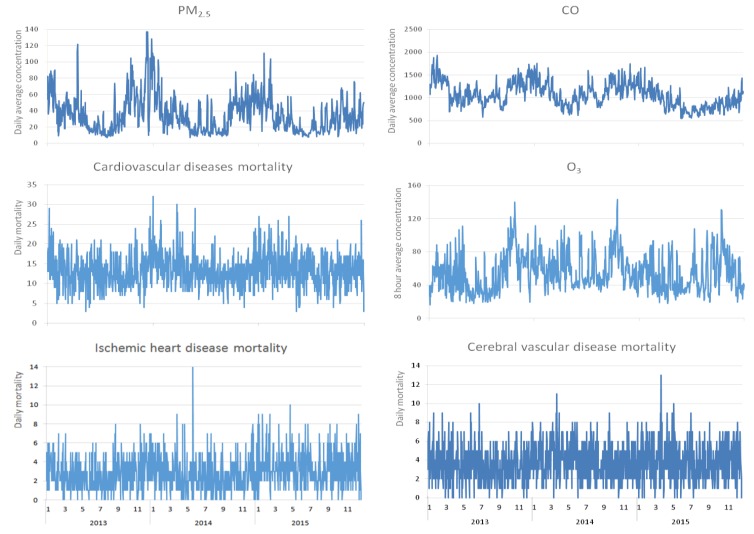
Time series chart on resident mortality versus air pollutant concentration in Shenzhen (2013–2015).

As revealed by the time series chart on resident mortality from CVD in Shenzhen versus air pollutant concentration in 2013–2015, the variation trend of CVD death and PM_2.5_ concentration was basically the same; both peaked in winter and spring, and both declined in summer and autumn. CO and O_3_ also have seasonal trends. See Figure 2 for details.

### 3.4. Correlation Analysis of Air Pollutants versus Meteorological Factors

According to the findings of the Spearman correlation analysis of air pollutants versus meteorological factors, among the six air pollutants, PM_2.5_, PM_10_, SO_2_, NO_2_, CO and O_3_ exhibited positive correlations between each pair (*p* < 0.05). The correlation between PM_2.5_ and PM_10_ was the strongest; their correlation coefficient was more than 0.9. Following that, the correlation between PM_2.5_ and SO_2_, and PM_2.5_ and NO_2_, were the next strongest. Their correlation coefficients were more than 0.6, respectively. The correlation between PM_2.5_ and CO, and PM_2.5_ and O_3_, were the weakest, and their correlation coefficients were 0.4 and 0.6, respectively. In terms of air pollutants versus meteorological factors, PM_2.5_, PM_10_, SO_2_ and NO_2_ each exhibited negative correlations with daily average temperature, daily average RH and daily average wind speed; PM_2.5_, PM_10_, SO_2_ and NO_2_ each exhibited positive correlations with daily average atmosphere pressure. A positive correlation was observed between daily average temperature and daily average RH (Table 3.)

### 3.5. Autocorrelation between Different Lags for PM*_2.5_*

There was autocorrelation between different lags of PM_2.5_. According to the findings of the autocorrelation between different lags for PM_2.5_, the correlation was strongest with a one day interval. The longer the interval, the weaker the correlation. The correlation coefficient was 0.825 at the maximum and 0.531 at the minimum. See Table 4 for details.

### 3.6. Analysis of GAM

For the effects of PM_2.5_ concentration on CVD mortality, IHD mortality and EVD mortality without controlling other pollutants and after controlling CO or O_3_, see Table 5.

#### 3.6.1. Effects of PM_2.5_ Concentration upon CVD Mortality

Without controlling other pollutants, PM_2.5_ concentration of lag0–lag5 on CVD mortality had significant effects. When PM_2.5_ concentration of lag0 to lag5 rose by 10 μg/m^3^, the excess risks of CVD mortality were 1.60% (95% CI: 0.48–2.73%), 1.40% (95% CI: 0.33–2.49%), 1.37% (95% CI: 0.33–2.42%), 1.00% (95% CI: −0.01–2.02%), 0.89% (95% CI: −0.11–1.90%) and 1.50% (95% CI: 0.51–2.50%), respectively. Of these, lag5 was the most significant (*p* = 0.003); lag3 and lag4 were marginally significant (*p* = 0.053; *p* = 0.081). The PM_2.5_ moving average concentration (lag01–lag04) on CVD mortality had significant effects. When PM_2.5_ concentration of lag01 to lag04 rose by 10 μg/m^3^, the ER of CVD mortality were 1.85% (95% CI: 0.63–3.09%), 2.09% (95% CI: 0.79–3.41%), 2.14% (95% CI: 0.79–3.51%) and 2.17% (95% CI: 0.78–3.59%), respectively. Of these, lag02 was the most significant (*p* = 0.0015), and all *P* were less than 0.01. After controlling CO, the results show that PM_2.5_ concentration of lag0–lag5 on CVD mortality still had significant effects. When PM_2.5_ concentration of lag0 to lag5 rose by 10 μg/m^3^, the ER of CVD mortality were 1.83% (95% CI: 0.54–3.13%), 1.65% (95% CI: 0.46–2.86%), 1.65% (95% CI: 0.51–2.82%), 1.51% (95% CI: 0.04–2.28%), 1.19% (95% CI: 0.09–2.31%) and 1.61% (95%CI: 0.52–2.72%), respectively. Of these, lag5 was still the most significant (*p* = 0.004). The effects maintain stability after controlling CO. The PM_2.5_ moving average concentration (lag01–lag04) on CVD mortality had significant effects. When PM_2.5_ concentration of lag01 to lag04 rose by 10 μg/m^3^, the ER of CVD mortality were 2.10% (95% CI: 0.75–3.48%), 2.37% (95% CI: 0.96–3.81%), 2.39% (95% CI: 0.93–3.86%) and 2.43% (95% CI: 0.93–3.95%), respectively. Of these, lag02 was the most significant (*p* = 0.0010), and all *P* were less than 0.01. The effects maintain stability after controlling CO. After controlling O_3_, the results show that PM_2.5_ concentration of lag0–lag5 on CVD mortality still had significant effects. When PM_2.5_ concentration of lag0 to lag5 rose by 10 μg/m^3^, the ER of CVD mortality were 1.63% (95% CI: 0.45–2.82%), 1.58% (95% CI: 0.41–2.76%), 1.55% (95% CI: 0.39–2.73%), 1.04% (95% CI: −0.10–2.20%), 1.25% (95% CI: 0.12–2.40%) and 2.28% (95% CI: 1.15–3.42%), respectively. Of these, lag5 was still the most significant (*p* = 0.000). The effects maintain stability after controlling O_3_. The PM_2.5_ moving average concentration (lag01–lag04) on CVD mortality had significant effects. When PM_2.5_ concentration of lag01 to lag04 rose by 10 μg/m^3^, the ER of CVD mortality were 2.04% (95% CI: 0.70–3.39%), 2.45% (95% CI: 0.98–3.93%), 2.56% (95% CI: 1.00–4.14%) and 2.77% (95% CI: 1.14–4.43%), respectively. Of these, lag02 was the most significant (*p* = 0.0010), and all *P* were less than 0.01. The effects maintain stability after controlling O_3_.

#### 3.6.2. Effects of PM_2.5_ Concentration upon IHD Mortality

Without controlling other pollutants, PM_2.5_ concentration of lag0 to lag2 and lag5 on IHD mortality had significant effects. When PM_2.5_ concentration of lag0 to lag2 and lag5 rose by 10 μg/m^3^, the ER of IHD mortality were 2.12% (95% CI: −0.18–4.47%), 2.92% (95% CI: 0.69–5.20%), 2.87% (95% CI: 0.71–5.07%) and 2.37% (95% CI: 0.33–4.46%), respectively. Of these, lag2 was the most significant (*p* = 0.009) and lag0 was marginally significant (*p* = 0.072). The PM_2.5_ moving average concentration (lag01–lag04) on IHD mortality had significant effects. When PM_2.5_ concentration of lag01 to lag04 rose by 10 μg/m^3^, the ER of IHD mortality were 3.51% (95% CI: 0.63–5.75%), 3.86% (95% CI: 1.17–6.63%), 3.60% (95% CI: 0.82–6.64%) and 3.58% (95% CI: 0.73–6.51%), respectively. Of these, lag02 was the most significant (*p* = 0.005). After controlling CO, the results show that PM_2.5_ concentration of lag0–lag5 on IHD mortality still had significant effects. When PM_2.5_ concentration of lag0 to lag5 rose by 10 μg/m^3^, the ER of IHD mortality were 3.26% (95% CI: 0.56–6.03%), 4.30% (95% CI:1.76–6.91%), 4.38% (95% CI: 1.93–6.88%), 2.01% (95% CI: −0.32–4.39%), 2% (95% CI: −0.30–4.35%) and 2.68% (95% CI: 0.39–5.03%), respectively. Of these, lag2 was still the most significant (*p* = 0.000) and lag3 and lag4 were marginally significant (*p* = 0.091; *p* = 0.089). The PM_2.5_ moving average concentration (lag01–lag04) on IHD mortality had significant effects. When PM_2.5_ concentration of lag01 to lag04 rose by 10 μg/m^3^, the ER of IHD mortality were 4.55% (95% CI: 1.67–7.50%), 5.39% (95% CI: 2.38–8.49%), 5.08% (95% CI: 2.01–8.25%) and 4.98% (95% CI: 1.84–8.22%), respectively. Of these, lag02 was the most significant (*p* = 0.000), and all *P* were less than 0.01. The effects maintain stability after controlling CO. After controlling O_3_, the results still show that PM_2.5_ concentration of lag0 to lag2 and lag5 on IHD mortality had significant effects. When PM_2.5_ concentration of lag0 to lag2 and lag5 rose by 10 μg/m^3^, the ER of IHD mortality were 2.62% (95% CI: 0.19–5.11%), 3.36% (95% CI: 0.93–5.85%), 3.14% (95% CI: 0.74–5.61%), 3.33% (95% CI: 0.98–5.73%), respectively. Of these, lag5 was still the most significant (*p* = 0.005). The PM_2.5_ moving average concentration (lag01–lag04) on IHD mortality had significant effects. When PM_2.5_ concentration of lag01 to lag04 rose by 10 μg/m^3^, the ER of IHD mortality were 3.85% (95% CI: 1.10–6.68%), 4.71% (95% CI: 1.68–7.83%), 4.34% (95% CI: 1.14–7.64%) and 4.55% (95% CI: 1.21–8.01%), respectively. Of these, lag02 was the most significant (*p* = 0.002), and all *P* were less than 0.01. The effects maintain stability after controlling O_3_.

#### 3.6.3. Effects of PM_2.5_ Concentration upon EVD Mortality

Without controlling other pollutants, PM_2.5_ concentration of lag0, lag1 and lag3 to lag5 on EVD mortality had significant effects. When PM_2.5_ concentration of lag0, lag1 and lag3 to lag5 rose by 10 μg/m^3^, the ER of EVD mortality were 2.03% (95% CI: 0.04–4.06%), 2.07% (95% CI: 0.16–4.02%), 1.90% (95% CI: 0.08–3.75%), 2.09% (95% CI: 2.28–3.92%) and 1.99% (95% CI: 0.20–3.81%), respectively. Of these, lag4 was the most significant (*p* = 0.023). The PM_2.5_ moving average concentration (lag01–lag04) on EVD mortality had significant effects. When PM_2.5_ concentration of lag01 to lag04 rose by 10 μg/m^3^, the ER of EVD mortality were 2.46% (95% CI: 0.32–4.64%), 2.52% (95% CI: 0.27–4.82%), 2.78% (95% CI: 0.45–5.16%) and 3.08% (95% CI: 0.68–5.53%), respectively. Of these, lag04 was the most significant (*p* = 0.012). After controlling CO, the results show that PM_2.5_ concentration of lag0, lag1 and lag3 to lag5 on EVD mortality still had significant effects. When PM_2.5_ concentration of lag0, lag1 and lag3 to lag5 rose by 10 μg/m^3^, the ER of EVD mortality were 2.16% (95% CI: −0.10–4.47%), 1.95% (95% CI: −0.14–4.10%), 1.78% (95% CI: −0.21–3.81%), 2.49% (95% CI: 0.50–4.51%) and 2.03% (95% CI: 0.07–4.04%), respectively. Of these, lag4 was still the most significant (*p* = 0.014) and lag0, lag1 and lag3 were marginally significant (*p* = 0.062; *p* = 0.068; *p* = 0.080). The PM_2.5_ moving average concentration (lag01–lag04) on EVD mortality had significant effects. When PM_2.5_ concentration of lag01 to lag04 rose by 10 μg/m^3^, the ER of EVD mortality were 2.43% (95% CI: 0.07–4.84%), 2.43% (95%CI: −0.01–4.93%), 2.63% (95%CI: 0.13–5.19%) and 2.99% (95% CI: 0.43–5.61%), respectively. Of these, lag04 was the most significant (*p* = 0.022). The effects maintain stability after controlling CO. The PM_2.5_ moving average concentration (lag02) was marginally significant (*p* = 0.051). After controlling O_3_, the results show that PM_2.5_ concentration of lag1 and lag3 to lag5 on EVD mortality had significant effects. When PM_2.5_ concentration of lag1 and lag3 to lag5 rose by 10 μg/m^3^, the ER of EVD mortality were 1.81% (95% CI: −0.29–3.96%), 2.09% (95% CI: 0.00–4.23%), 1.83% (95% CI: −0.25–3.95%), 1.83% (95% CI: −0.23–3.94%), respectively. Of these, lag3 was still the most significant (*p* = 0.049) and lag1, lag4 and lag5 were marginally significant (*p* = 0.092; *p* = 0.085; *p* = 0.083). The PM_2.5_ moving average concentration (lag01–lag04) on EVD mortality had still significant effects. The effects maintain stability after controlling O_3_. When PM_2.5_ concentration of lag01 to lag04 rose by 10 μg/m^3^, the ER of EVD mortality were 2.06% (95% CI: −0.28–4.47%), 2.43% (95% CI: −0.14–5.06%), 2.82% (95% CI: 0.09–5.62%) and 2.97% (95% CI: 0.12–5.91%), respectively. Of these, lag04 was the most significant (*p* = 0.041). The PM_2.5_ moving average concentration (lag01and lag02) were marginally significant (*p* = 0.085; *p* = 0.064).

See Figure S1 for RR and 95% CI of mortality per 10 μg/m3 increase in PM2.5 concentration with different lags 0–5 days prior to mortality (lag0–lag5) and moving averages from day 0 to day prior to mortality (lag01–lag04).

### 3.7. Concentration–Response Relationship

**Figure 3:**
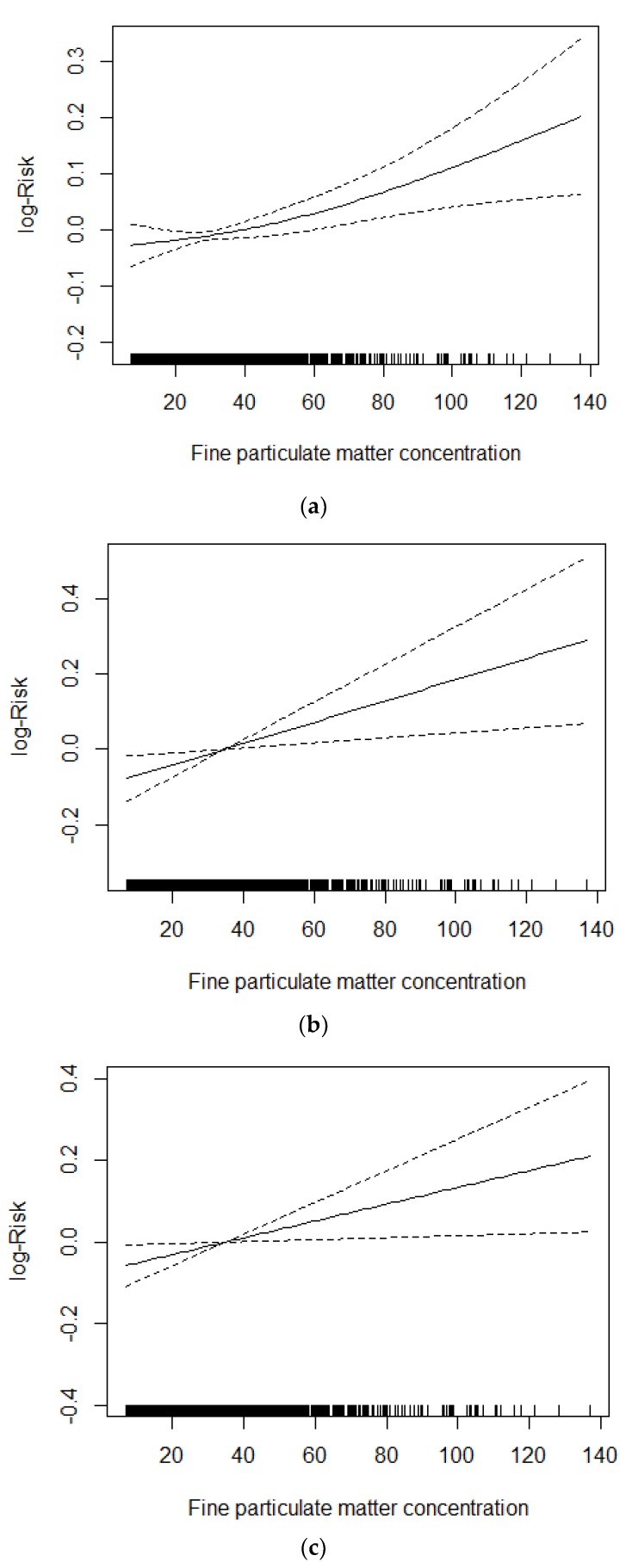
(**a**) Association between daily lag5 cardiovascular disease mortality and PM_2.5_ concentration (μg/m^3^) considered in the generalized additive model (GAM). (**b**) Association between daily lag2 IHD mortality and PM_2.5_ concentration (μg/m^3^) considered in the GAM. (**c**) Association between daily lag4 EVD mortality and PM_2.5_ concentration (μg/m^3^) considered in the GAM.

In each single-day lag (lag0–lag5), the strongest effect of PM_2.5_ concentration upon CVD mortality, IHD mortality and EVD mortality were chosen to make concentration–response relationship figures. See Figure 3 for the concentration–response relationship between PM_2.5_ daily average concentration and CVD mortality, IHD mortality and EVD mortality with different lags of PM_2.5_ concentration. According to the figure, based on the control of long-term and seasonal trends of resident mortality, effect of day of the week, effect of public holidays, daily average temperature and daily average RH, the relative risk (RR) of CVD mortality, IHD mortality and EVD mortality increases along with the increase in PM_2.5_ daily average concentration.

## 4. Discussion

This study aims to explore the association between PM_2.5_ and CVD mortality, IHD mortality and EVD mortality in areas with lighter air pollution in China. The results show that elevated PM_2.5_ concentration can increase the risk of CVD mortality, IHD mortality and EVD mortality of residents. There are also lag effects. The effects maintain stability after controlling other pollutants (CO, O_3_). The results of this study are consistent with the meta-analysis results of previous literature on the relationship between air pollution and CVD mortality [5,23]. Epidemiological studies have demonstrated a consistent increased risk for cardiovascular events in relation to both short-term and long-term exposure to present-day concentrations of ambient particulate matter [35].

The putative biological mechanisms linking air pollution to heart disease involve direct effects of pollutants on the cardiovascular system, blood, and lung receptors, and/or indirect effects mediated through pulmonary oxidative stress and inflammatory responses. Direct effects may occur via agents that readily cross the pulmonary epithelium into the circulation, and possibly along with soluble constituents of PM_2.5_. In addition, activation of pulmonary neural reflexes secondary to PM interactions with lung receptors may play a role. Ensuing alterations in autonomic tone, under appropriate circumstances, might contribute to the instability of a vascular plaque or initiate cardiac arrhythmias. These direct effects of air pollution represent a plausible explanation for the occurrence of rapid cardiovascular responses, such as increased myocardial infarctions. Chronic indirect effects may occur via pulmonary oxidative stress/inflammation induced by inhaled pollutants. This may subsequently contribute to a systemic inflammatory state, which may in turn be capable of activating hemostatic pathways, impairing vascular function, and accelerating atherosclerosis [35].

PM_2.5_ and daily mortality in 272 Chinese cities from 2013 to 2015 at two levels of nationwide and six geographic regions were studied by Chen et al. The article mainly analyzed two day moving averages of PM_2.5_ concentrations. The results showed that each 10 mg/m^3^ increase in the two day moving average of PM_2.5_ concentrations were significantly associated with increments in mortality of 0.27% from cardiovascular diseases and 0.23% from stroke. Furthermore, the associations were stronger in cities with lower PM_2.5_ levels or higher temperatures [9].

In our study, PM_2.5_ concentration of lag0–lag5 on CVD mortality had significant effects without controlling other pollutants. When PM_2.5_ concentration of lag0 to lag5 rose by 10 μg/m^3^, the ER of CVD mortality were 1.60%, 1.40%, 1.37%, 1.00%, 0.89% and 1.50%, respectively. Of these, lag5 was the most significant, and lag3 and lag4 were marginally significant. According to the meta-analysis results of Atkinson, et al., a 10 μg/m^3^ increment in PM_2.5_ was associated with a 0.84% increase in the risk of CVD mortality in the world [5]. The result is lower than the results of our study. Atkinson et al. also reported that the risk of CVD mortality in America, Europe and the Western Pacific region increased by 0.84%, 2.26% and 0.56%, respectively [5]. The results of our study are between the Americas and European (higher than the results of the study in the Americas and Western Pacific region, lower than European countries). Shenzhen has lighter air pollution and higher temperatures. Chen et al. reported that each 10 mg/m^3^ increase in two day moving averages of PM_2.5_ concentrations was significantly associated with increments in mortality of 0.27% from cardiovascular diseases nationwide [9]. Our study shows that when PM_2.5_ concentration of lag02 rose by 10 μg/m^3^, the ER of CVD mortality was 2.09% in Shenzhen. This result is lower than the results of our study. Our findings prove Chen et al.’s discovery. Dai et al. reported a 1.03% increase in CVD deaths in association with a 10 μg/m^3^ increase in two day averaged PM_2.5_ concentration [23]. In our study, when PM_2.5_ concentration of lag02 rose by 10 μg/m^3^, the ER of CVD mortality was 2.09%. Our results are higher than the results of Dai et al. Maté et al. reported that for each increase of 10 μg/m^3^ in daily mean PM_2.5_ concentration, the relative risks (RR) were as follows: for overall circulatory mortality, associations were established at lags 2 and 6, with a RR of 1.022 and 1.025 respectively in Madrid, Spain [36]. The results are similar to the results of our study. Kan et al. reported that PM_2.5_ concentration lag0 and moving average concentration lag01 on cardiovascular disease mortality had significant effects in Shanghai, China. A 10 μg/m^3^ increase in the lag01 concentration of PM_2.5_ corresponded to 0.41% as the strongest effect increase of cardiovascular disease mortality [24]. It is lower than the results of our study (2.09%). Beelen et al. reported an analysis of 22 European cohorts. For PM_2.5_, the hazard ratio deaths from cardiovascular disease was 1.21 per 5 μg/m^3^ [37]. Chen et al. reported that stronger associations between PM_2.5_ exposure and mortality from cardiovascular disease hazard ratio (HR) = 1.35 in Canada [38]. Pinault et al. reported an analysis of the Canadian community health survey cohort that each 10 μg/m^3^ increase in exposure was associated with increased risks of circulatory disease mortality HR = 1.19 [39]. Cesaroni et al. reported that the strongest association was found for cardiovascular disease mortality (HR = 1.06 per 10 μg/m^3^ PM_2.5_) in Rome [26]. Wong et al. reported that cardiovascular disease mortality hazard ratios per 10 μg/m^3^ increase in PM_2.5_ was 1.22 in residents older than 65 years in Hong Kong [40]. Zanobett and Schwartz conducted a national, multi-city time-series study of the acute effect of PM_2.5_ on the increased risk of death for CVD for the years 1999–2005. They found a 0.85% increase in CVD deaths for a 10 μg/m^3^ increase in two day averaged PM_2.5_ [41].

In our study, PM_2.5_ concentration of lag0 to lag2 and lag5 on IHD mortality had significant effects without controlling other pollutants. While PM_2.5_ concentration of lag0 to lag2 and lag5 rose by 10 μg/m^3^, the ER of IHD mortality were 2.12%, 2.92%, 2.87% and 2.37%, respectively. Of these, lag2 was the most significant (*p* = 0.009). According to the data meta-analysis results of Atkinson et al., a 10 μg/m^3^ increment in PM_2.5_ was associated with a 3.36% increase in the risk of IHD mortality in the world [5]. The result is higher than the results of our study. Jerrett et al. used a subset of the American Cancer Society (ACS) cohort (Los Angeles, CA, USA) to estimate a within-city RR of 1.49 per 10 μg/m^3^ increase in PM_2.5_ [42]. The results are lower than the results of our study. Xie et al. reported that PM_2.5_ concentration was significantly associated with IHD mortality in Beijing [43]. Our study shows that PM_2.5_ concentration was marginally significantly associated with IHD mortality. Xie et al. reported that a 10 μg/m^3^ increase in PM_2.5_ was associated with a 0.27% increase in IHD mortality on the same day [43]. The result of our study shows that a 10 μg/m^3^ increase in PM_2.5_ was associated with a 2.12% increase in IHD mortality on the same day. Chen et al. reported that associations between PM_2.5_ exposure and IHD mortality HR = 1.43 in Canada [38]. Pinault et al. reported that each 10 μg/m^3^ increase in exposure was associated with increased risks of IHD mortality HR = 1.290 [39]. Cesaroni et al. reported that the strongest association was found for IHD mortality (HR = 1.10 per 10 μg/m^3^ PM_2.5_) in Rome [26]. Wong et al. reported that IHD mortality hazard ratios per 10 μg/m^3^ increase in PM_2.5_ were 1.42 in residents older than 65 years Hong Kong [40]. Maté et al. reported that for every increase of 10 μg/m^3^ in daily mean PM_2.5_ concentration, no statistically significant association was found with other IHD mortality in Madrid, Spain [36]. Maté et al.’s result is different from the results of our study. Thurston et al.’s research shows that associations with IHD mortality varied by PM_2.5_ mass constituent and source [27].

In our study, PM_2.5_ concentration of lag0, lag1 and lag3 to lag5 on EVD mortality had significant effects without controlling other pollutants. While PM_2.5_ concentration of lag0, lag1 and lag3 to lag5 rose by 10 μg/m^3^, the ER of EVD mortality were 2.03%, 2.07%, 1.90%, 2.09% and 1.99%, respectively. Of these, lag4 was the most significant. The PM_2.5_ moving average concentration (lag01–lag04) on EVD mortality had significant effects. When PM_2.5_ concentration of lag01 to lag04 rose by 10 μg/m^3^, the ER of EVD mortality were 2.46%, 2.52%, 2.78% and 3.08%, respectively. Of these, lag04 was the most significant. According to the meta-analysis by Wan et al. from 1966–2014 literature, the results show that a 10 μg/m^3^ increase in PM_2.5_ was associated with a 1.4% increase in EVD mortality [44]. The result is lower than the results of our study. Chen et al. reported that each 10 mg/m^3^ increase in two day moving average of PM_2.5_ concentrations was significantly associated with increments in mortality of 0.23% from stroke nationwide [9]. Our study shows that when PM_2.5_ concentration of lag02 rose by 10 μg/m^3^, the ER of EVD mortality were 2.52% in Shenzhen. Our results confirmed the discovery by Chen et al. that the associations between PM_2.5_ and daily mortality were stronger in cities with lower PM_2.5_ levels or higher temperatures [9]. Pinault et al. reported an analysis of the Canadian community health survey cohort that each 10 μg/m^3^ increase in exposure was associated with increased risks of EVD mortality HR = 1.241 (significant HR *p >* 0.05) [39]. Wong et al. reported that EVD mortality hazard ratios per 10 μg/m^3^ increase in PM_2.5_ was 1.24 in residents older than 65 years in Hong Kong [40]. Maté et al. reported that for every increase of 10 μg/m^3^ in daily mean PM_2.5_ concentration, no statistically significant association was found with EVD mortality in Madrid, Spain [36]. Maté et al.’s result is different from the results of our study.

The time series chart showed that the variation trend of cardiovascular disease mortality counts and PM_2.5_ concentration basically matched. According to the concentration–response relationship figures, the RR of cardiovascular disease mortality, IHD mortality and EVD mortality increased along with the increase of PM_2.5_ daily average concentration. The time series chart and the concentration–response relationship figures all indicate that PM_2.5_ has an effect on the death from cardiovascular disease of the residents.

The average age of death from cardiovascular disease was 67.4 years old in Shenzhen during 2013–2015; the average age of IHD death was 66.6 years old; the average age of EVD death was 66.0 years old. The results are younger than China’s average life expectancy (the average life expectancy of China’s population is 74.8 years old) [45]. The reason for this may be that although Shenzhen belongs to the economically developed area, there is a higher standard of living, but Shenzhen is a city of immigrants. Many people come from all over the country to live and work in Shenzhen, these people are mainly young, so the residents of Shenzhen’s actual age is younger than that in other parts of China. This is also one of the reasons why the annual mortality rate in Shenzhen is lower than that in other parts of China. The death rate is 2.4 per thousand in 2014 in Shenzhen, and China is 7.2 per thousand in 2014 [46,47].

Reported results at different places in the world are not exactly the same, which may be related to the following factors. First, the main source of pollutants, pollutant concentration and composition of PM_2.5_ are different in different areas and different periods. Ambient particulate matter is a heterogeneous mixture of various compounds (e.g., organic and elemental carbon, metals, sulfates, nitrates, and microorganisms) from multiple sources (e.g., traffic, manufacturing, power generation) [44]. Second, different methods were used to assess residents’ exposure to pollutants. Third, the control confounding factors in the process of study are not completely the same. Fourth, the definitions of ICD coding of diseases classification are different. Fifth, the study populations have different demographic characteristics. Sixth, the meteorological factors of research in different areas and different periods are different. Seventh, small study biases exist in studies of cardiovascular disease mortality [5]. Reasons for heterogeneity in effect estimates in different regions of the world require further investigation [5,44].

## 5. Strengths and Limitations

In this study, data on resident mortality were collected from Shenzhen CDC, Shenzhen Public Security Bureau and Shenzhen Funeral Home, which represent deaths of local residents. This study analyzed the data of 2013–2015 for three consecutive years, and the results were stable. In this study, several factors may affect the mortality of residents, such as long-term trend, seasonal trend, day of the week, public holiday, daily average temperature and daily average relative humidity, were controlled in the model. The model objectively analyzes the relationship between PM_2.5_ concentration and total cardiovascular disease mortality, PM_2.5_ concentration and IHD mortality, PM_2.5_ concentration and EVD mortality.

The present study also has some limitations. The local-specific factors that could affect the association between pollutant exposure and mortality such as PM chemical composition, existing emission sources, socioeconomic factors, etc. are not incorporated in the study and need to be researched further.

## 6. Conclusions

Although the mean of PM_2.5_ daily average concentration was 35.0 μg/m^3^ in Shenzhen, showing a slight pollution in China, PM_2.5_ still increased the amount of cardiovascular disease, IHD and EVD mortality. Therefore, it is recommended that residents need to use personal protection, especially the residents with cardiovascular disease and in pollution weather, thereby reducing the adverse effects of PM_2.5_ on health. The government still needs to strengthen the governance of air pollution in areas with lighter air pollution.

## Figures and Tables

**Table 1:** Basic information of death residents from cardiovascular disease (CVD) in Shenzhen (2013–2015).

Category	Total Deaths	Percent Among Total Deaths (%)	Daily Average Number of Deaths	Standard Deviation	Lowest Daily Deaths	Q1	Q2	Q3	Highest Daily Deaths
Total death	41,815	100.0	38.2	7.6	10	33	38	43	77
CVD death	14,537	34.8	13.3	4.1	3	11	13	16	32
IHD death	3188	7.6	2.9	1.8	0	2	3	4	14
EVD death	4028	9.6	3.7	2.0	0	2	4	5	13

Q1, Q2, Q3 are equal to the 25%, 50% and 75% number of all values in the sample arranged from small to large, respectively. CVD = cardiovascular disease. IHD = ischemic heart disease. EVD = cerebral vascular disease.

**Table 2:** Air pollutants and meteorological factors in Shenzhen (2013–2015).

Indicator	Mean	Standard Deviation	Minimum	Q1	Q2	Q3	Maximum	No. of Days of Exceeding Grade 1 Criterion *	No. of Days of Exceeding Grade 2 Criterion **
Air pollutants									
PM_2.5_ (μg/m^3^)	35.0	21.9	7.1	17.4	29.9	47.1	137.1	458	58
PM_10_ (μg/m^3^)	55.6	30.2	10.9	31.4	47.7	71.6	181.8	513	11
SO_2_ (μg/m^3^)	10.4	4.8	3.5	7.4	9.2	11.9	54.8	1	0
NO_2_ (μg/m^3^)	43.8	17.5	14.7	31.9	39.7	51.6	133.7	47	47
CO (μg/m^3^)	1061.9	259.7	542.9	857.1	1034.1	1239.1	1930.4	0	0
O_3_ (μg/m^3^)	55.2	22.3	16.4	37.6	50.8	70.0	143.3	42	0
Meteorological factors									
Daily average temperature (°C)	23. 5	5.5	6.0	19.2	24.8	28.2	33.0		
Daily average RH (%)	73.3	13.3	19.0	67.0	75.0	82.0	100.0		
Daily average atmosphere pressure (kpa)	1005.6	6.4	986.8	1000.7	1005.5	1010.8	1019.9		
Daily average wind speed (m/s)	2.1	0.8	0.3	1.5	2.0	2.5	5.5		

Q1, Q2, Q3 are equal to the 25%, 50% and 75% number of all values in the sample arranged from small to large, respectively. * The number of days for average daily concentration exceeded the national Grade 1 criterion of China. ** The number of days for average daily concentration exceeded the national Grade 2 criterion of China. The formula for calculating the standard deviation of pollutants is as follows: $\sigma =\sqrt{\frac{1}{N}\sum_{i=1}^{N}{\left(x_{i}-\mu \right)}^{2}}$ Standard Deviation is represented by *σ*. *x_i_* represents the average concentration of seven air quality monitoring sites on a given day. *µ* indicates the daily average concentration of seven air quality monitoring sites in three years. *N* = 1095. PM_2.5_ = particulate matter with a particle size below 2.5 microns. PM_10_ = particulate matter with particle size below 10 microns. RH = relative humidity.

**Table 3:** Spearman correlation analysis of air pollutants and meteorological factors in Shenzhen during 2013–2015 (r value).

Items	PM_10_	SO_2_	NO_2_	CO	O_3_	Daily Average Temperature	Daily Average RH	Daily Average Atmosphere Pressure	Daily Average Wind Speed
PM_2.5_	0.956	0.622	0.628	0.447	0.567	−0.556	−0.515	0.595	−0.111
PM_10_		0.680	0.628	0.399	0.601	−0.447	−0.591	0.529	−0.127
SO_2_			0.612	0.257	0.318	−0.176	−0.515	0.270	−0.143
NO_2_				0.343	0.063 *	−0.415	−0.118	0.346	−0.400
CO					0.189	−0.315	−0.075*	0.242	0.037n
O_3_						−0.117n	−0.572	0.240	0.078 *
Daily average temperature (°C)							0.200	−0.858	−0.093
Daily average RH (%)								−0.451	−0.046n
Daily average atmosphere pressure (kpa)									0.047n

*p* < 0.01, except * *p* < 0.05 and n *p* > 0.05.

**Table 4:** Correlation coefficient of different lags for PM_2.5_ (μg/m^3^).

Items	lag1	lag2	lag3	lag4	lag5
lag0	0.825	0.692	0.631	0.582	0.531
lag1		0.825	0.692	0.631	0.583
lag2			0.824	0.691	0.631
lag3				0.825	0.691
lag4					0.824

PM_2.5_ concentration with different lags from 0 to 5 days prior to mortality (lag0–lag5).

**Table 5:** The effects of PM_2.5_ concentration on CVD, IHD and EVD mortality without controlling other pollutants and controlling CO or O_3_ in Shenzhen (2013–2015).

Items	Single Pollutant Model (Without Controlling Other Pollutants)				2-Pollutant Model (Controlling CO)				2-Pollutant Model (Controlling O_3_)			
	*β*	StdErr	*P*	ER% (95% CI)	*β*	StdErr	*P*	ER% (95% CI)	*β*	StdErr	*P*	ER (95% CI)
Cardiovascular disease mortality												
lag0	0.0016	0.0006	0.005 #	1.60 (0.48–2.73)	0.0018	0.0006	0.005 #	1.83 (0.54–3.13)	0.0016	0.0006	0.007 #	1.63 (0.45–2.82)
lag1	0.0014	0.0005	0.010 *	1.40 (0.33–2.49)	0.0016	0.0006	0.006 #	1.65 (0.46–2.86)	0.0016	0.0006	0.008 #	1.58 (0.41–2.76)
lag2	0.0014	0.0005	0.010 *	1.37 (0.33–2.42)	0.0016	0.0006	0.005 #	1.65 (0.51–2.82)	0.0015	0.0006	0.009 #	1.55 (0.39–2.73)
lag3	0.0010	0.0005	0.053 @	1.00 (−0.01–2.02)	0.0011	0.0006	0.042 *	1.15 (0.04–2.28)	0.0010	0.0006	0.074 @	1.04 (−0.1–2.2)
lag4	0.0009	0.0005	0.081 @	0.89 (−0.11–1.90)	0.0012	0.0006	0.034 *	1.19 (0.09–2.31)	0.0012	0.0006	0.031 *	1.25 (0.12–2.4)
lag5	0.0015	0.0005	0.003 #	1.50 (0.51–2.50)	0.0016	0.0006	0.004 #	1.61 (0.52–2.72)	0.0023	0.0006	0.000 #	2.28 (1.15–3.42)
lag01	0.0018	0.0006	0.003 #	1.85 (0.63–3.09)	0.0021	0.0007	0.002 #	2.1 (0.75–3.48)	0.0020	0.0007	0.003 #	2.04 (0.7–3.39)
lag02	0.0021	0.0007	0.002 #	2.09 (0.79–3.41)	0.0023	0.0007	0.001 #	2.37 (0.96–3.81)	0.0024	0.0007	0.001 #	2.45 (0.98–3.93)
lag03	0.0021	0.0007	0.002 #	2.14 (0.79–3.51)	0.0024	0.0007	0.001 #	2.39 (0.93–3.86)	0.0025	0.0008	0.001 #	2.56 (1–4.14)
lag04	0.0021	0.0007	0.002 #	2.17 (0.78–3.59)	0.0024	0.0008	0.001 #	2.43 (0.93–3.95)	0.0027	0.0008	0.001 #	2.77 (1.14–4.43)
Ischemic heart disease mortality												
lag0	0.0021	0.0012	0.072 @	2.12 (−0.18–4.47)	0.0032	0.0014	0.018 *	3.26 (0.56–6.03)	0.0026	0.0012	0.035 *	2.62 (0.19–5.11)
lag1	0.0029	0.0011	0.010 *	2.92 (0.69–5.20)	0.0042	0.0013	0.001 #	4.3 (1.76–6.91)	0.0033	0.0012	0.006 #	3.36 (0.93–5.85)
lag2	0.0028	0.0011	0.009 #	2.87 (0.71–5.07)	0.0043	0.0012	0.000 #	4.38 (1.93–6.88)	0.0031	0.0012	0.010 *	3.14 (0.74–5.61)
lag3	0.0011	0.0011	0.289	1.12 (−0.94–3.24)	0.0020	0.0012	0.091 @	2.01 (−0.32–4.39)	0.0008	0.0012	0.487	0.84 (−1.5–3.23)
lag4	0.0014	0.0010	0.179	1.41 (−0.64–3.50)	0.0020	0.0012	0.089 @	2 (−0.3–4.35)	0.0019	0.0012	0.104	1.94 (−0.4–4.34)
lag5	0.0023	0.0010	0.023 *	2.37 (0.33–4.46)	0.0026	0.0012	0.022 *	2.68 (0.39–5.03)	0.0033	0.0012	0.005 #	3.33 (0.98–5.73)
lag01	0.0031	0.0013	0.014 *	3.15 (0.63–5.75)	0.0044	0.0014	0.002 #	4.55 (1.67–7.5)	0.0038	0.0014	0.006 #	3.85 (1.1–6.68)
lag02	0.0038	0.0013	0.005 #	3.86 (1.17–6.63)	0.0053	0.0015	0.000 #	5.39 (2.38–8.49)	0.0046	0.0015	0.002 #	4.71 (1.68–7.83)
lag03	0.0035	0.0014	0.011 *	3.60 (0.82–6.45)	0.0050	0.0015	0.001 #	5.08 (2.01–8.25)	0.0042	0.0016	0.008 #	4.34 (1.14–7.64)
lag04	0.0035	0.0014	0.014 *	3.58 (0.73–6.51)	0.0049	0.0016	0.002 #	4.98 (1.84–8.22)	0.0045	0.0017	0.007 #	4.55 (1.21–8.01)
Cerebral vascular disease mortality												
lag0	0.0020	0.0010	0.045 *	2.03 (0.04–4.06)	0.0021	0.0011	0.062 @	2.16 (−0.1–4.47)	0.0017	0.0011	0.121	1.67 (−0.44–3.81)
lag1	0.0020	0.0010	0.034 *	2.07 (0.16–4.02)	0.0019	0.0011	0.068 @	1.95 (−0.14–4.1)	0.0018	0.0011	0.092 @	1.81 (−0.29–3.96)
lag2	0.0015	0.0009	0.126	1.46 (−0.40–3.36)	0.0013	0.0010	0.199	1.34 (−0.7–3.42)	0.0017	0.0011	0.108	1.74 (−0.38–3.91)
lag3	0.0019	0.0009	0.040 *	1.90 (0.08–3.75)	0.0018	0.0010	0.080 @	1.78 (−0.21–3.81)	0.0021	0.0011	0.049 *	2.09 (0–4.23)
lag4	0.0021	0.0009	0.023 *	2.09 (0.28–3.92)	0.0025	0.0010	0.014 *	2.49 (0.5–4.51)	0.0018	0.0011	0.085 @	1.83 (−0.25–3.95)
lag5	0.0020	0.0009	0.029 *	1.99 (0.20–3.81)	0.0020	0.0010	0.043 *	2.03 (0.07–4.04)	0.0018	0.0010	0.083 @	1.83 (−0.23–3.94)
lag01	0.0024	0.0011	0.024 *	2.46 (0.32–4.64)	0.0024	0.0012	0.043 *	2.43 (0.07–4.84)	0.0020	0.0012	0.085 @	2.06 (−0.28–4.47)
lag02	0.0025	0.0011	0.028 *	2.52 (0.27–4.82)	0.0024	0.0012	0.051 @	2.43 (−0.01–4.93)	0.0024	0.0013	0.064 @	2.43 (−0.14–5.06)
lag03	0.0027	0.0012	0.019 *	2.78 (0.45–5.16)	0.0026	0.0013	0.039 *	2.63 (0.13–5.19)	0.0028	0.0014	0.043 *	2.82 (0.09–5.62)
lag04	0.0030	0.0012	0.012 *	3.08 (0.68–5.53)	0.0029	0.0013	0.022 *	2.99 (0.43–5.61)	0.0029	0.0014	0.041 *	2.97 (0.12–5.91)

# *p* < 0.01; * *p* < 0.05; @ *p* < 0.10. StdErr = standard error. ER = excess risk.

## Supplementary Materials

The following are available online at http://www.mdpi.com/1660-4601/15/9/1918/s1, 1. Analysis of GAM (Figure S1. RR and 95% CI of mortality per 10 μg/m^3^ increase in PM_2.5_ concentration with different lags 0–5 days prior to mortality (lag0–lag5) and moving averages from day 0 to day prior to mortality (lag01–lag04)). 2. People’s Republic of China Ministry of Environmental Protection, Atmospheric environmental quality standards (GB3095-2012) (Table S1).
