# Supplementary material for: Association between Airborne Fine Particulate Matter and Residents’ Cardiovascular Diseases, Ischemic Heart Disease and Cerebral Vascular Disease Mortality in Areas with Lighter Air Pollution in China
Source: Int J Environ Res Public Health. 2018 Sep 3;15(9):1918. doi: 10.3390/ijerph15091918 (PMC6164472; doi:10.3390/ijerph15091918)
Supplement: Supplementary file 1 [file ijerph-15-01918-s001.pdf]

International Journal of
Environmental Research and Public Health

Supplementary Material

Association between Airborne Fine Particulate Matter and Residents' Cardiovascular Diseases, Ischemic Heart Disease and Cerebral vascular Disease Mortality in Area with Lighter Air Pollution in China

Junfang CAI [1,^], Shuyuan YU [2,^], Yingxin PEI [3], Chaoqiong PENG [2], Yuxue LIAO [2], Ning LIU [2], Jia Jia JI [2], Jinquan CHENG [2,*]

3.5. Analysis of GAM

Based on the control of long-term trend, seasonal trend, effect of DOW, effect of PH of residents' mortality, and daily average temperature and daily average RH, single pollutant model (no adjustment of pollutants) was used to calculate the RR value and its 95%CI lag0-lag5, lag01-lag04 of $PM_{2.5}$ daily average concentration versus residents' mortality from CVD. The results show positive correlation between $PM_{2.5}$ daily average concentration with lag0-lag5, lag01-lag04 and CVD mortality; positive correlation between $PM_{2.5}$ daily average concentration with lag0, lag1, lag2, lag5, lag01-lag04 and IHD mortality; positive correlation between $PM_{2.5}$ daily average concentration with lag0, lag1, lag3, lag4, lag5, lag01-lag04 and EVD mortality (see Figure S1-1). After controlling CO or O_3 separately, the results of double-pollutant models show positive correlation between $PM_{2.5}$ daily average concentration with lag0-lag5, lag01-lag04 and CVD mortality. The results were consistent with no adjustment of CO and O_3. The effects maintain stability after controlling CO or O_3. After controlling CO, the results show positive correlation between $PM_{2.5}$ daily average concentration with lag0-lag5, lag01-lag04 and IHD mortality; positive correlation between $PM_{2.5}$ daily average concentration with lag0, lag1, lag3, lag4, ag5, lag01-lag04 and EVD mortality (see Figure S1-2). After controlling O_3, the results show positive correlation between $PM_{2.5}$ daily average concentration with lag0, lag1, lag2, lag5, lag01-lag04 and IHD mortality; positive correlation between $PM_{2.5}$ daily average concentration with lag1, lag3, lag4, lag5, lag01-lag04 and EVD mortality (see Figure S1-3).

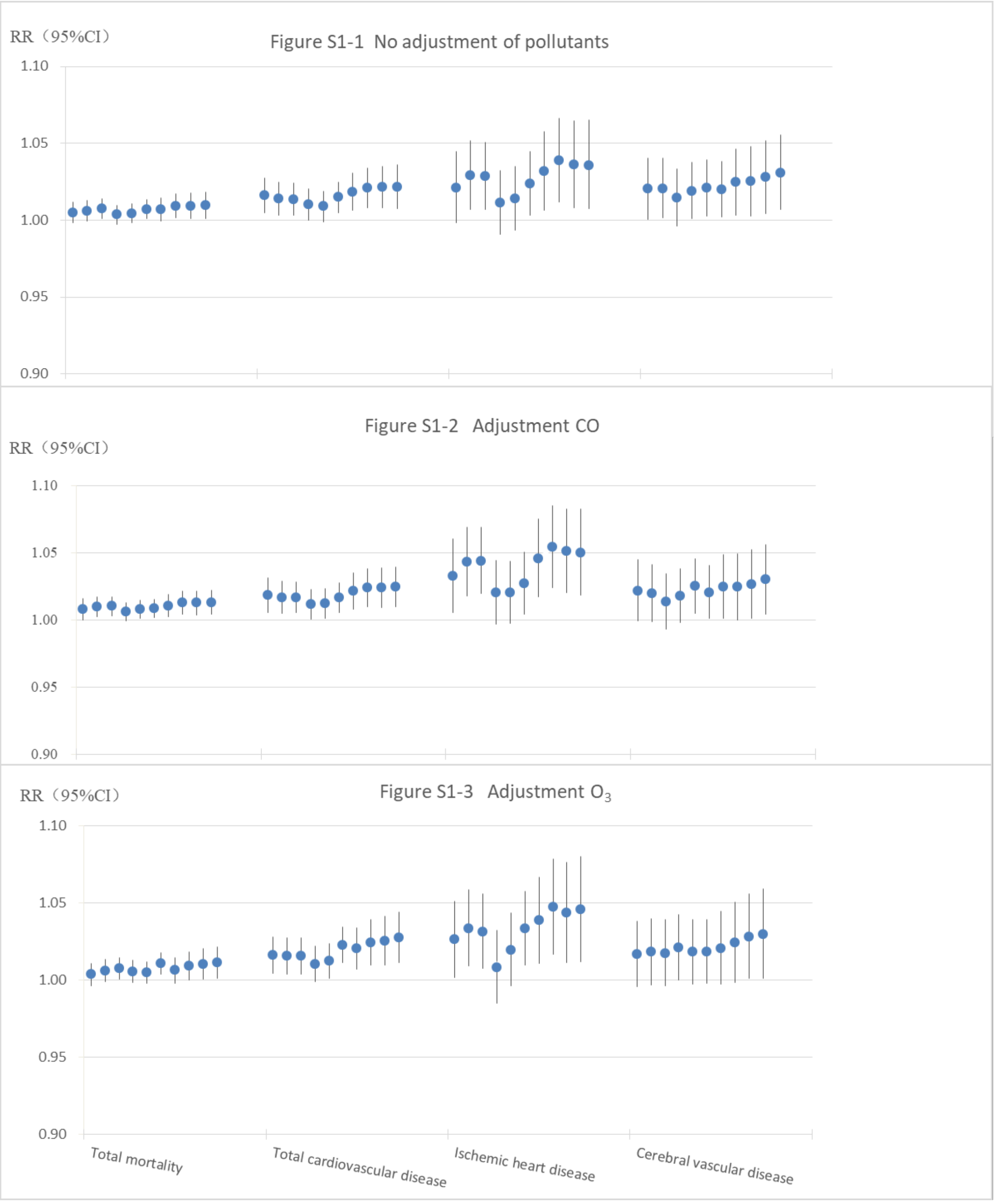

Figure S1. RR and 95% CI of mortality per 10μg/m^3 increase in $PM_{2.5}$ concentration with different lags 0–5 days prior to mortality (lag0–lag5) and moving averages from day 0 to day prior to mortality (lag01–lag04).

Table S1. People's Republic of China Ministry of Environmental Protection, Atmospheric environmental quality standards (GB3095-2012)

**Pollutant**	**Mean Time**	**Concentration Limit**		**Unit**
		grade1 criterion	grade2 criterion	
SO_2	Annual mean	20	60	μg/m^3
	24-hour average	50	150	
	1-hour average	150	500	
NO_2	Annual mean	40	40	μg/m^3
	24-hour average	80	80	
	1-hour average	200	200	
CO	24-hour average	4000	4000	μg/m^3
	1-hour average	10000	10000	
O_3	8-hour average	100	160	μg/m^3
	1-hour average	160	200	
PM_{10}	Annual mean	40	70	μg/m^3
	24-hour average	50	150	
$PM_{2.5}$	Annual mean	15	35	μg/m^3
	24-hour average	35	75	

According to < People's Republic of China Ministry of Environmental Protection, Atmospheric environmental quality standards (GB3095-2012) >, Natural reserves, scenic spots and other areas requiring special protection apply grade1 criterion. Residential areas, mixed residential areas for commercial transportation, cultural areas, industrial areas and rural areas apply grade2 criterion.
